# Self-regulated critical brain dynamics originate from high frequency-band activity in the MEG

**DOI:** 10.1371/journal.pone.0233589

**Published:** 2020-06-11

**Authors:** Stefan Dürschmid, Christoph Reichert, Nike Walter, Hermann Hinrichs, Hans-Jochen Heinze, Frank W. Ohl, Giulio Tononi, Matthias Deliano

**Affiliations:** 1 Department of Behavioral Neurology, Leibniz Institute for Neurobiology, Magdeburg, Germany; 2 Department of Neurology, Otto-von-Guericke University, Magdeburg, Germany; 3 Forschungscampus STIMULATE, Otto-von-Guericke University, Magdeburg, Germany; 4 CBBS—center of behavioral brain sciences, Otto-von-Guericke University, Magdeburg, Germany; 5 Research Section Applied Consciousness Science, Department of Psychosomatic Medicine, University Medical Center, Regensburg, Germany; 6 German Center for Neurodegenerative Diseases (DZNE), Magdeburg, Germany; 7 Dept. Systems Physiology of Learning (SPL), Leibniz Institute for Neurobiology (LIN), Magdeburg, Germany; 8 Institute of Biology (IBIO), Otto-von-Guericke University, Magdeburg, Germany; 9 Department of Psychiatry, University of Wisconsin-Madison, Madison, Wisconsin, United States of America; 10 Center for sleep and consciousness, University of Wisconsin-Madison, Madison, Wisconsin, United States of America; Dalhousie University, CANADA

## Abstract

Brain function requires the flexible coordination of billions of neurons across multiple scales. This could be achieved by scale-free, critical dynamics balanced at the edge of order and disorder. Criticality has been demonstrated in several, often reduced neurophysiological model systems. In the intact human brain criticality has yet been only verified for the resting state. A more direct link between the concept of criticality and oscillatory brain physiology, which is strongly related to cognition, is yet missing. In the present study we therefore carried out a frequency-specific analysis of criticality in the MEG, recorded while subjects were in a defined cognitive state through mindfulness meditation. In a two-step approach we assessed whether the macroscopic neural avalanche dynamics is scale-free by evaluating the goodness of a power-law fits of cascade size and duration distributions of MEG deflections in different frequency bands. In a second step we determined the closeness of the power-law exponents to a critical value of -1.5. Power-law fitting was evaluated by permutation testing, fitting of alternative distributions, and cascade shape analysis. Criticality was verified by defined relationships of exponents of cascade size and duration distributions. Behavioral relevance of criticality was tested by correlation of indices of criticality with individual scores of the Mindful Attention Awareness Scale. We found that relevant scale-free near-critical dynamics originated only from broad-band high-frequency (> 100 Hz) MEG activity, which has been associated with action potential firing, and therefore links criticality on the macroscopic level of MEG to critical spike avalanches on a microscopic level. Whereas a scale-free dynamics was found under mindfulness meditation and rest, avalanche dynamics shifted towards a critical point during meditation by reduction of neural noise. Together with our finding that during mindfulness meditation avalanches show differences in topography relative to rest, our results show that self-regulated attention as required during meditation can serve as a control parameter of criticality in scale-free brain dynamics.

## Significance statement

Our study bridges the gap between criticality, brain physiology and cognition. We show that scale-free critical dynamics in the MEG can be observed in the broad-band high-frequency (>100 Hz) activity that has been associated with action potential firing. Our study provides a link between critical avalanche dynamics at a macroscopic MEG and spike avalanches at a microscopic level. We show that mindfulness meditation shifts scale-free dynamics towards the critical point by reducing neural noise. In contrast to a state of rest, mindful focused attention requires detection and inhibition of mind-wandering, and the refocusing of breath, and therefore relies on constant monitoring and executive control, particularly in novices. This could be implemented by brain states balanced at an instable critical point between order and disorder allowing for flexibly focusing and shifting attention. Self-regulated attention might thereby serve as a control parameter of criticality in the scale-free brain dynamics.

## Introduction

The proper functioning of the human brain rests on the electrical activity of billions of neurons coordinated across multiple scales. Theoretical and experimental work in physics has shown that the multi-scale dynamics of a complex system can be characterized by the spatial and temporal statistics of avalanches branching through the system. These statistics reveal whether the system is in a fully random or a fully ordered state, or whether it is in a critical state, i.e. in a complex state at the edge between order and disorder [1,2]. Empirically, critical avalanche dynamics in neuronal networks has been first demonstrated in cell cultures and slices in vitro by the seminal work of [3]. Further in vitro studies have shown that at the critical point, network functions are optimized with respect to the susceptibility of inputs, dynamic range of input/output relationships, information transmission, and information capacity [4,5], neural networks can become highly flexible, display meta-stable patterns, and adapt more easily to different rules through Hebbian plasticity [6]. Thus, information processing functions would be optimized if the network dynamics operates at its critical point [7].

However, being a concept derived from physics, criticality still needs to be empirically linked to cognitive brain functions in awake subjects, more directly. Different markers of criticality like long-range correlation in spontaneous low frequency (10 and 20Hz) EEG activity in humans have been studied [8]. In humans, criticality of neural avalanches comparable to unit firing propagation has only been demonstrated reliably for the resting state condition. Shriki et al. [9] showed that macroscopic whole brain avalanches defined by peaks and troughs of broad-band MEG activity in awake humans at rest have no characteristic scale, typical for a system state close to a critical point. However, the physiological nature of neural avalanches on a macroscopic level remains unclear. Since peaks and troughs in the MEG/EEG can reflect different phases of spatially and temporally extended oscillatory generators, reconstruction of avalanches on a macroscopic level should take into account the polarity and the frequency of the brain signals to disentangle superimposed sources. Most importantly, the functional relevance of criticality and its role in cognition is still an open question.

Most experimental studies in awake healthy subjects have been carried out under resting conditions, as task related activity implies non-stationarity and superposition of stimulus- and response-driven activities [10], for which current statistical analyses of criticality are not well suited. However, human subjects can be verbally instructed to constantly alter their resting state dynamics through self-regulation switching into a different, stationary operational mode. A prominent self-regulation technique is mindfulness meditation, which we hypothesize is purportedly suited to induce changes in criticality, i.e. how close the system is as to its critical point. During meditation, mindful focused attention (MFA) is required to maintain focus on sensations over an extended period of time, which reduces distractor noise while at the same time cognitive control is required to detect phases of mind wandering [11–14] during which attention is directed elsewhere, as it occurs during an uninstructed resting state. In fact, mindfulness (meditation) and mind wandering (resting state) can be considered as opposing stationary background states, mediated by attentional subnetworks and the default mode network, respectively [15]. Irrmischer et al. [16] hypothesized that focused attention is balanced at a critical point of instability between order and disorder allowing for both, transient focus and swift change of attentional resources. They have shown that criticality is reduced in trained practitioners, i.e. when the meditation task becomes a habit. This would suggest that during MFA in contrast to the resting state, brain dynamics is tuned closer to a critical point. However, Irrmischer et al. show [16] that criticality is reduced in trained practitioners during MFA compared to rest. In trained practitioners, meditation might therefore require less effort, which could explain the observed shift towards a subcritical dynamics, or, as suggested by other studies, trained practitioners might experience difficulties in refraining from meditation practice during rest [17]. Thus, it is important to differentiate state from trait changes [18,19]. In our study we combined a frequency and polarity specific analysis of the macroscopic neural avalanche dynamics with a variation of the internal cognitive state by comparing a group of subjects performing mindfulness meditation with a group under rest. To avoid the aforementioned possible confounds between meditation-induced state and trait changes, we employed a simple mindful breathing task in novices. As human resting-state activity displays criticality [9], and MFA enhances attentional control [14,20], we hypothesize that top-down attention–in contrast to bottom-up attention [10]–modulation during mindfulness training tunes neural networks closer towards the critical point. Specifically, since it is assumed that MFA and resting activity are mediated by different neuronal networks [21–23], we further hypothesize that spatio-temporal avalanche dynamics will involve different cortical regions in these states.

## Methods

### Participants and paradigm

**Fig 1:**
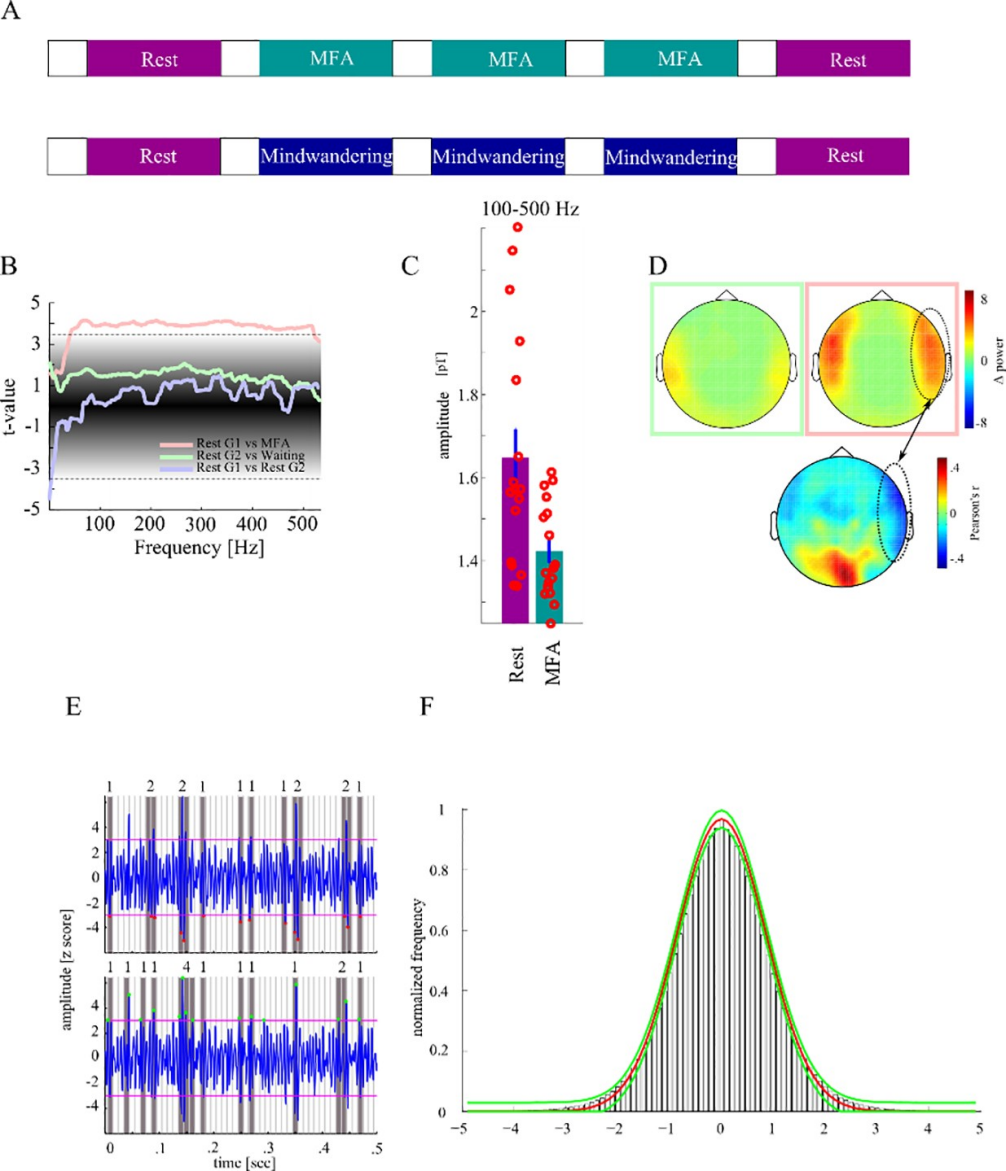
Procedure of the experiment. ***A*** The mindful focused attention (MFA) experiment conducted with group 1 consisted of five blocks each 5 min long and initiated by an instruction either to rest or to meditate with the breath as the primary object of awareness while in intermediate blocks in group 2 short stories were read from the same speaker and subjects had to wait for 5 min afterwards. ***B*** Power spectral density was compared between rest, mindful focused attention and in the mind-wandering condition of group 1 (G1) and group 2 (G2), respectively. Colored lines show difference in power values (t-values) as a function of frequency, within each group between the first resting block, and the MFA- (pink line), W- (green line) and final resting blocks (violet line), respectively. The black shaded area gives the surrogate distribution against which each t-value was compared. The horizontal lines give the confidence interval. ***C*** We observed a significant decrease of power between rest and MFA across a wide frequency spectrum covering the gamma and high frequency activity range. ***D*** shows the topographical distribution of differences in power in the high frequency band. The green and pink square correspond with the green and pink line in ***C***, respectively. MEG magnetometers showing the strongest difference in power were located bilaterally over a fronto-temporal region. The lower panel shows correlation of individual MAAS scores with power difference between rest and MFA. Only in sensors covering the right frontal cortex we found both power difference between rest and MFA and correlation of these power differences with MAAS score. ***E*** shows MEG activity with trough (upper panel) and peak (lower panel) events showing different patterns of clustering yielding different likelihood distribution of cascade sizes. ***F*** shows Gaussian fit to histogram of trough and peak events (red line mean, green line lower and upper confidence interval of estimated Gaussian fits.

Seventeen healthy participants (eleven females; mean age 28.3#x00B1;7.5 SD years, two left-handed), with no history of neurological disorders participated in group MFA. MEG activity was recorded while subjects were at rest or had to apply MFA (**Fig 1A**). Specifically, subjects started with a resting period, followed by an instructed meditation, and finally were asked to rest again without MFA. The whole experiment consisted of a sequence of five blocks: Rest (5min)–MFA (5min)–MFA (5min)–MFA (5min)–Rest (5min). Each block was initiated by the instruction either to rest or to apply MFA. Instructions (see S1 Data) were recorded in advance and spoken by an experienced meditator (extensive training in Vipassana-meditation [24] with an overall of 2500 hours of experience during a time period of 5 years and prior experience as a meditation teacher). In sum, preceding the first MFA phase, subjects were instructed to concentrate on the breathing cycle as the primary object of awareness. Following the first MFA block another audio file was played, reminding the participants to refocus on their breathing whenever they caught their mind wandering. Then the second MFA phase started. Thereafter a third audio file was presented as a reminder to continue with the task of focussing on the breathing cycle. A third MFA phase lasting another 5 min was recorded that ended with another audio stimulus to initialize a following measurement of resting state activity for 5 minutes. The participants kept their eyes closed during the whole procedure; the volume of the audio stimuli was adjusted individually before the MEG recording to a comfortable level. Another group of ten subjects with no history of neurological disorders forming the mind wandering (MW) control group (mean age 26±2.75 SD years, one left-handed) carried out the same experiment with the difference that instead of listening to the MFA instruction, short stories (excerpts from the book “Let me tell you a story: Tales along the road to happiness” written by Jorge Bucay) were read by the same speaker (**Fig 1B**). After each short story participants were asked to wait until the next one to be narrated. In essence, we recorded five blocks of rest, which is characterized by mind-wandering. For a better comparison with group 1 we tagged intermediate blocks as blocks of MW: Rest (5min)–MW (5min)–MW (5min)–MW (5min)–Rest (5min). Additionally, the trait Mindful Attention Awareness Scale (MAAS [25]), a 15-item scale designed to assess a core characteristic of mindfulness, was answered by all subjects of both groups. Higher scores reflect higher levels of dispositional mindfulness. All participants gave their written informed consent prior to recordings and were compensated financially for their time. MAAS scores were compared between groups with an unpaired t-test. The study was approved by the local ethics committee (“Ethical Commitee of the Otto-von-Guericke University Magdeburg”).

### Data acquisition

The subjects were seated in a magnetically shielded room in which magnetoencephalographic activity (MEG) was recorded while the subjects performed the experiment. Electrooculographic (EOG) activity was recorded to reconstruct vertical and horizontal eye movements. Electrode impedance was kept below 10 kΩ. For data acquisition a whole-head, 102-sensor element array (Elekta Neuromag® TRIUX^TM^) was used. Each of the102 sensor elements was equipped with one magnetometer measuring the normal field component and two orthogonally oriented planar gradiometers for measuring the gradient components. The participants sat in an upright position underneath the MEG “helmet”. MEG data were sampled at 2000 Hz and low-pass filtered with 660 Hz cutoff frequency.

### Preprocessing

We used Matlab 2013b (Mathworks, Natick, USA) for all offline data processing. Only magnetometers were involved in our analyses. All filtering (see below) was done using zero phase-shift IIR filters (filtfilt.m in Matlab). First, we used an absolute threshold of 300 fT to discard signal epochs of excessive, non-physiological amplitude. We then visually inspected all data, excluded epochs exhibiting excessive muscle activity, as well as time intervals containing artifactual signal distortions, such as signal steps or pulses. We refrained from applying artifact reduction procedures that affect the dimensionality and/or complexity of the data like independent component analysis.

### Analysis

We estimated the power spectral density (PSD) for different blocks and groups. Specifically, for each subject we calculated PSD as a function of frequency for the first and the last rest block across the entire 5 min interval using Welch's method based on the FFT [26]. Afterwards, the spectra were averaged yielding one mean spectrum for each subject of group 1 for the rest condition. The same was done for the three blocks of MFA. In the second group the power spectra of both blocks of resting were similarly calculated and averaged. Then the power spectra of all 3 MW blocks were calculated and averaged. Across the two groups this yields four PSD frequency spectra: rest of group 1, MFA of group 1, rest of group 2, and MW of group 2. In planned comparisons we tested (i) whether the PSD (coefficient of each frequency) differed between groups in the rest block, (ii) MFA differs from rest within group 1, and (iii) MW from rest within group 2. We hypothesized that rest does not differ between both groups while potentially MFA results in an altered PSD compared to rest. Hence, for each frequency we conducted three t-tests using log power values: rest vs. MFA within group 1, rest vs. MW within group 2, and rest group 1 vs. rest group 2. To correct the significance threshold for multiple comparisons resulting t-values were compared against a single surrogate distribution which was constructed by randomly reassigning labels (i.e. rest group 1, rest group 2, MFA, MW) to the values of subjects in 10,000 runs [27]. In each iteration we randomly picked two (out of four) labels and assigned randomly the values of subjects. This results in 10,000 surrogate t-values from which we determined a two-sided 99.9% confidence interval as significance threshold.

### Phase spectral analysis of peaks and troughs in the broad-band signal

We then analyzed whether the local minima of negative excursions (troughs) or local maxima of positive excursions (peaks) of the broad-band signal are sufficiently sensitive events to detect certain phases of oscillations in narrow frequency bands (see **Fig 1E** for difference between troughs–local minima in negative excursions–and peaks–local maxima in positive excursions). Hence, we asked whether events represent only certain phases at *certain* frequencies, or certain phases of *all* frequencies. This is important if peaks and troughs of the broad-band signal are actually generated by band-limited processes. We tested this in the following way. The raw signal was filtered between 1 and 275 Hz. A notch filter was applied to remove line noise (±2Hz around the first 5 harmonics).

We then discretized the broad-band signal largely following the procedure proposed by [9]. Epochs in which amplitudes exceeded ± 5 SD were marked as artefacts and excluded. Then, the time series of all blocks in group 1 were z-transformed individually for all five blocks (rest 1, MFA1, MFA2, MFA3, rest 2), separately for each magnetometer. Positive and negative excursions exceeding the chosen threshold of ±3 SDs [9] from the mean for each magnetometer were identified (see **Fig 1E & 1F**). A single peak event was identified as local maximum within each positive excursion, and a single trough event as local minimum within each negative excursion. We extracted cascades across our set of MEG magnetometers by first binning the whole time series in 10 ms windows which is the average bin duration in previous studies [9] and then summing up all events found within each bin for each magnetometer.

Peak time points (TP_peak_) and trough time points (TP_trough_) were stored. We then filtered the broad-band signal in 39 narrow frequency bands with exponentially spaced center-frequencies between 6 and 250 Hz each with a bandwidth of 10% (IIR Filter of order 4) around the center-frequency. Note that frequency bands around line noise and harmonics can be assessed since filtered frequency bands are broader than notch filters applied. In each frequency we extracted the instantaneous phase angle (Hilbert function in Matlab). Both, at all TP_peak_ and TP_trough_ we estimated the phase of each of 39 narrow frequencies. This results in two phase angle distributions for each narrow frequency band–one at the time point of the broad-band troughs and one at the broad-band peaks. For each distribution, we calculated the concentration coefficient κ (reciprocal value to variance).

$$\kappa =\frac{1}{\varsigma^{2}}$$

across all phase angles in each frequency band. A low κ represents an equal likelihood of all possible phases (-π to +π), which means that the phase of specific frequency is unrelated to the peak or trough event. A higher κ, on the other hand indicates that, a certain phase is overrepresented within the phase distribution, i.e. that the oscillation at the corresponding frequency is time- and phase-locked to the broad-band peak or trough event. To determine significance, we compared frequency specific κ values against a surrogate distribution. In 10,000 runs we draw 10,000 random phase values from the phase angle distributions across all frequency and calculated surrogate κ values and determined the 99% confidence interval of the resulting surrogate distribution.

To investigate criticality features in the MEG data, we performed the following analysis steps. In short, we first assessed spectral differences between the experimental conditions rest, MFA and MW within and between groups. In the next step we analyzed whether peaks and troughs in the broad-band signal are associated with certain phases of oscillations in narrower frequency bands. Then we carried out a first frequency- and polarity-specific analysis of criticality across all conditions by fitting a truncated power-law to each cascade size and duration distribution, and by assessing the closeness of its exponent α to a critical value of α = -1.5. We thereby systematically follow a two-step approach: Before interpreting the exponent, we always assessed the goodness of the power-law fit, as an insufficient goodness of fit would leave the estimated exponent uninterpretable. The frequency specific analysis of criticality allowed us to determine candidate frequency bands of the critical dynamics. Then we carried out a more detailed analysis of criticality with MEG signals filtered in these candidate bands, including comparisons of alternative fitting distributions, shuffling tests, evaluation of exponent relationships between cascade size and duration distributions, a shape analysis of temporal cascade profiles, as well as a correlation analysis between individual power-law fits (exponents and residuals) and MAAS scores. This initial analysis then allowed us to specifically analyze changes in criticality with experimental conditions by statistically comparing goodness of power-law fit and exponents within and between groups under conditions of MFA, MW, and rest. Finally, we assessed the topographical origin of the branching dynamics, and across which cortical regions cascades extend during rest vs. MFA to assess similarity and overlap of the involved networks.

### Frequency-specific analysis of criticality

In the next step we assessed whether the brain exhibits criticality and if this is a frequency-specific phenomenon. We assessed this in the following way as outlined in [3,9]. We filtered the broadband signal in 39 narrow frequency bands with exponentially spaced center-frequencies between 6 and 250 Hz, each with a bandwidth of 10% (IIR Filter of order 4) around the center-frequency in each of the five blocks. We then discretized the bandpass filtered signal in the same way as the broad-band signal (see above, and **Fig 1E & 1F**). Separately for peak and trough events, we extracted cascades across our set of MEG magnetometers by first binning the whole time series in 10 ms windows which is the average bin duration in previous studies [9] and then summing up all events found within each bin for each magnetometer. The same analysis was repeated for the frequency bands but different bin durations ranging from 5 to 20 ms. A cascade was defined as a continuous sequence of time bins in which there was an event on any magnetometer, ending with a time bin with no events on any magnetometer. The sum of events across all magnetometers in a cascade was defined as the cascade size as it has been described in previous studies [3,9]. To avoid double dipping in the selection of frequency bands displaying criticality, we did not make use of differences in meditative state for selecting the bands. Thus, cascades were pooled across all blocks of a group including both rest and MFA/MW conditions. Using a power mass function we determined the likelihood of each cascade size (CS) within the set of all cascades. The exponent of the likelihood distribution of the cascade size (CSLD) was determined by the slope of a linear regression line fitted to the log-log representation of the CSLD in each subject. Here we used the first 2/3 of each single log-log CSLD to exclude the sharply dropping likelihood for the longest cascades.

The residuals of the linear fit to the CSLD quantify the deviation of the CSLD from power-law scaling, and thus from a critical regime. In each frequency band we compared the residuals for trough cascades with the residuals for the peak cascades by calculating t-values across subjects.

To correct the significance threshold for multiple comparisons, the resulting 39 t-values were individually compared against the same surrogate distribution [27]. In 10,000 runs we randomly assigned the residual of our participants to peak and trough cascades. This yields a surrogate distribution of 10,000 t-values for each frequency band. We then estimated the probability of each observed t-value of each frequency band within the surrogate distribution of the given frequency band. For assessing the frequency characteristics of criticality, we constructed the surrogate distribution of CSLD exponents in the following way. We randomly permuted CSLDs in each subject and for each frequency, and estimated the slopes of their log-log representation, again. The dispersion of the surrogate distribution was used to determine a confidence interval around a hypothesized exponent of α = -1.5, which according to theoretical considerations indicates critical systems behavior [9]. Frequency bands with a mean CSLD exponent α across subjects lying within the confidence interval were considered showing critical behavior since it did not deviate from theoretically assumed criticality parameter (α = -1.5). As further control, time series of each magnetometer was shifted in time separately by a randomly chosen number of samples (N_permutations_ = 10,000) leading to new cascades with unsystematic spatial spread. Both, an exponent closer to -1.5 and/or smaller residuals in the original compared to the surrogate data would be indicative for criticality in the original data.

In addition to linear regression, we tested whether cascade size distributions can be better described by an exponential fit or a log-normal fit, which would indicate a different non-critical systems state. To this end we compared residuals of the linear fit with residuals of the exponential fit and log-normal fit. T-values were compared against a surrogate distribution. This surrogate distribution was constructed in the following way. In 10,000 runs we randomly swapped labels (linear vs. exponential vs. log-normal) and calculated new t-values yielding a surrogate distribution of 10,000 t-values against which we compared the observed t values.

Additionally, we compared the ratio of the exponents of the cascade size distribution (α) and cascade duration distribution (τ) with the exponent ρ of the cascade size vs. cascade duration distribution [28]. The ratio ρ´ is given by $\frac{\alpha -1}{\tau -1}$. We compared ρ and ρ´ values in paired t tests both for the LFB and the HFB. In a critical state, the relation ρ is not different from ρ´ [28].

### Avalanche shape collapse

If a neural system is in a critical state, in addition to exhibiting power-law size and duration distributions, the mean temporal profiles of avalanches should be identical across scales [28,29] meaning that long duration avalanches should have the same scaled mean shape as short avalanches (shape collapse). Shapes were produced by averaging the temporal profiles (number of events in consecutive time bins) of all avalanches of a particular duration. We then scaled shapes to a uniform duration and scaled the minimal and maximal numbers of events constituting a cascade between 0 and 1.

### External validity

To assess the behavioral relevance of criticality, we tested whether mindfulness correlates with the goodness of the linear fit in the critical frequency bands as an indicator of power-law scaling and thus of criticality. For this, we correlated the residuals with the summed MAAS scores.

### Differences between mindful focused attention and rest

The previous analysis informed us which frequency bands show criticality. We then filtered the signals in the frequency bands (to anticipate, a low frequency (9-37Hz) and a high frequency band (170-275Hz) showing exponents close to criticality α = -1.5). Separately for the three blocks of MFA and the two blocks of resting activity, we discretized the resulting bandpass filtered signal and extracted the cascades as outlined above. Then we estimated the CSLD for both conditions and compared exponents in planned comparisons with a t-test. In the second step we separately determined the CSLD exponent for each of the five blocks of group 1 to test the specific hypotheses that *(i)* brain dynamics were regulated towards criticality from the first block (rest) to the second block (mindful focused attention), *(ii)* remained at a constant critical level throughout the following two blocks of mindful focused attention and *(iii)* rebound from critical dynamics in the last block when mindful focused attention is suspended (rest). Each t-value resulting from the pairwise comparisons were compared against a surrogate distribution to test for significance. In 10,000 runs we randomly assigned condition labels (rest vs. MFA) to the CSLD exponent values across subjects and calculated a t-value between rest and MFA from these surrogate data. This yields 10,000 new t-values against which we compared the observed t-values. Finally, we tested whether waiting and rest differed in the control group comparing exponent values with a paired t-test.

### Topographic analysis of avalanche propagation

We further characterized the topographic spread of the avalanches. First, we evaluated where cascades typically started and hence most likely triggered the avalanches. This was assessed in the frequency bands showing criticality according to the previous analysis steps. To this end, we determined the likelihood of each magnetometer to show an event in the first time bin of each cascade. We did this only for cascades longer than 10 time bins. We hypothesized that shorter cascades cannot spread fast enough allowing for a comparison between trigger zones on the one hand and regions where cascades typically propagate on the other hand. If cascades systematically spread away from the trigger zone then this is most likely detectable in longer cascades. The likelihood of each magnetometer in the first time bin yielded a topographic distribution of avalanche starting points, separately for rest and MFA in each subject. In each MEG magnetometer we compared the likelihood during rest and MFA across subjects with a t-test yielding a t-value for each MEG magnetometer. Regions of MEG magnetometers with a significant negative t-value mark those regions where cascades during MFA originate from more frequently. Conversely, regions of MEG magnetometers with a significant positive t-value denote those regions where cascades originate more often during rest than during MFA. Second, to reveal cortical regions across which cascades extend, we determined for each cascade all MEG magnetometers involved. Then we calculated for each MEG magnetometer the likelihood to be involved in any cascade. This was done for each subject both in the MFA and the rest condition. The likelihood of each MEG magnetometer to be involved was compared between MFA and rest across subjects yielding a t-value for each MEG magnetometer. Regions of MEG magnetometers with a significant negative t-value mark those regions where cascades during MFA frequently originate from. To correct the significance threshold for multiple comparisons, t-values of each MEG sensor were compared against a surrogate distribution. In 10,000 runs we randomly assigned condition labels (rest vs. MFA) to the CSLD exponent values across subjects and calculated a t-value between rest and MFA from these surrogate data. This yields 10,000 new t-values against which we compared the observed t-values.

## Results

### MAAS score for trait mindfulness

The two experimental groups did not differ with respect to the score of the trait Mindful Attention Awareness Scale (MAAS: M_group1_ = 4.32; SD = 0.49; M_group2_ = 4.41; SD = 0.56; t_25_ = .44; p = 0.7). Thus, potential differences in brain dynamics are not due to different levels of mindfulness.

### Spectral power differences

We compared PSD spectra between rest, MFA and MW in planned comparisons to assess whether these conditions differ regarding oscillatory activity (**Fig 1B**). We found a difference in PSD within group 1 between rest and MFA from 42 to 520 Hz as indicated by t-values exceeding the upper boundary of the 99.9% confidence interval of a surrogate distribution (ci_99.9_ = [-3.37 3.48]) corresponding to a decrease of high frequency activity during MFA compared to rest (**Fig 1C**). The maximal t-value was 4.16 (p = .00004) at 72 Hz. In contrast, we did not find significant differences between rest and MW within the second group, nor in the rest condition between group 1 and group 2. MEG magnetometers showing the strongest difference in power were located bilaterally over a fronto-temporal region (**Fig 1D**, *upper panels*). Furthermore, individual MAAS scores were significantly correlated with a high frequency power decrease in a right set of fronto-temporal magnetometers and an increase in mid-occipital magnetometers (**Fig 1D**, *lower panel*) indicating that activity modulation in these regions predicts subjective mindfulness. In summary, we found a selective decrease in power of high frequency activity during MFA (**Fig 1C*)***. The comparison with the MW control condition (**Fig 1B**) suggests that this modulation of the high frequency activity was not due to changes in arousal over recording time but was truly related to meditation.

### Neural oscillations associated with broad-band peaks and troughs

**Fig 2:**
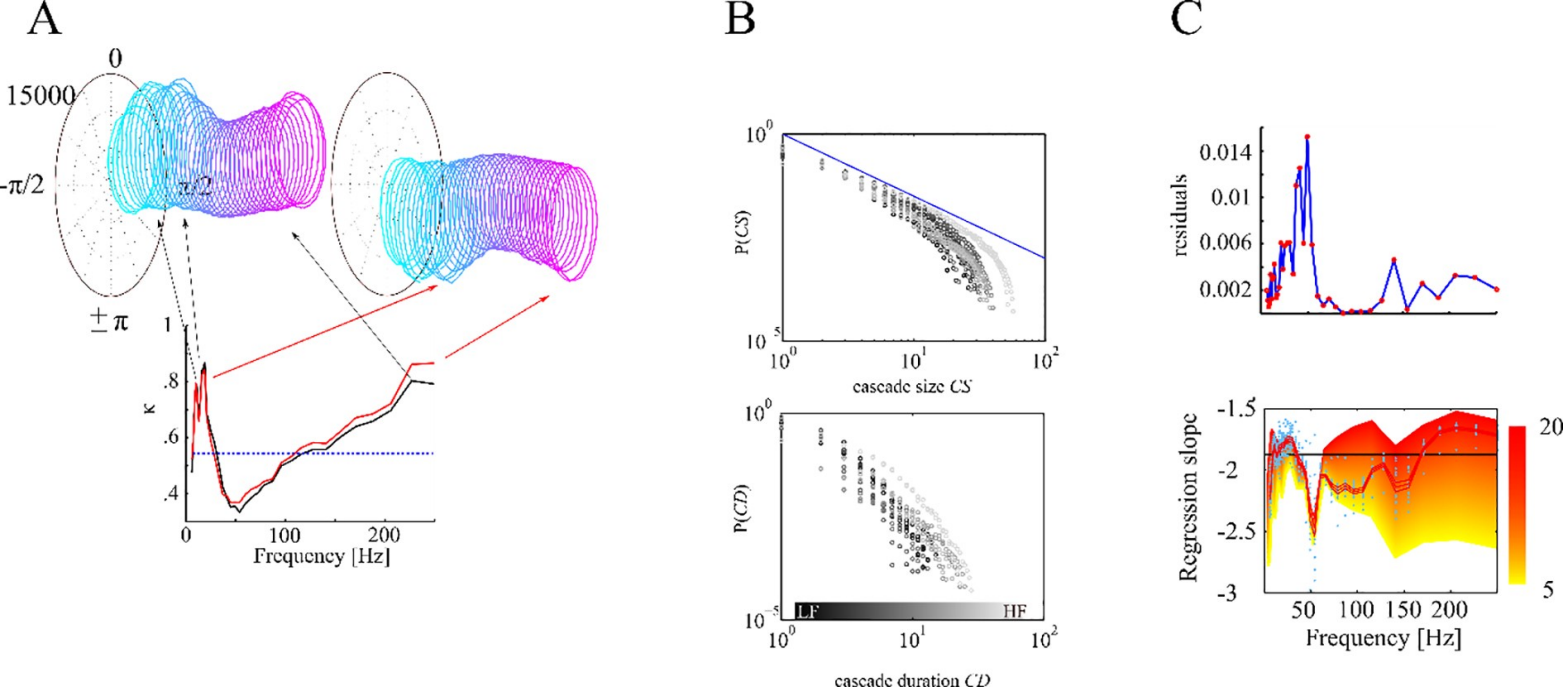
Depiction of trough and peak cascades. ***A*** We extracted the peaks (black) and troughs (red) of the broad band signal. At these time points we estimated the phase distribution of all 39 narrow frequency bands. Each ring represents the phase distribution of one frequency band ranging from low (cyan) to high frequencies (pink). We calculated the phase concentration κ for each of the frequencies (lower panel). The phase distributions in the alpha, beta, gamma and high-frequency range have an oval (corresponding with high κ) while frequencies around 50 Hz have more circular form (corresponding with low κ). ***B*** shows cascade size and cascade duration likelihood distributions for one subject for the different frequency bands in a log-log representation. Low frequencies are shown in darker shades. ***C*** We found systematically lower residuals (better linear fit) for 9–37 Hz (LFB) and 170–275 Hz (HFB) frequency bands and slopes not different from the critical value α = -1.5. Red line shows average across subjects (individual values shown by blue dots) and standard errors for 10 ms time bins.

In previous studies, neural avalanches have been defined on the basis of troughs and peaks in the broad-band signal of magnetometers, which have been used as basic events for reconstructing avalanches [3,9]. However, the broad-band signal can be split into narrow frequency bands which are associated with different psycho-physiological processes. By phase spectral analysis we determined, whether peaks and troughs in the broad-band signal were associated with certain phase angle of band-limited oscillations. Particularly, instantaneous phase angle at the time point of each peak or trough event were determined by a Hilbert transform of 39 narrow frequency band signals (exponentially spaced center-frequencies between 6 and 250 Hz each with a bandwidth of 10%) obtained by digitally filtering the broad-band signal (IIR Filter of order 4). Phase angle distributions were calculated across all TP_peak_ and TP_trough_ of all conditions in the experimental group, respectively, separately for each of the 39 narrow frequency bands. From these distributions, concentration κ (reciprocal value to standard deviation) was calculated for peaks and troughs in each frequency band, respectively. Phase distributions in the *alpha*, *beta*, *gamma* (LFB: low frequency band) and *high-frequency* range (HFB: high frequency band) have a biased oval while frequencies around 50 Hz have more symmetric, circular form (**Fig 2A**) and showed significant κ values (κ_crit_ = .55; LFB: κ_max_ = .87, p < .0001; HFB: κ_max_ = .88, p < .0001). Thus, peaks and troughs were associated with specific oscillatory phases of low-frequency oscillations (*alpha*, *beta*, *gamma*) and of high-frequency activity, but not of mid-frequency oscillations.

### Frequency bands displaying critical brain dynamics

To assess whether criticality has to be considered as a global or a band-limited phenomenon, we investigated the frequency-characteristics of the critical avalanche dynamics by fitting truncated power-laws to cascade size distributions derived from narrow-band filtered MEG signals (**Fig 2B and 2C**). Whether the dynamics is scale-free can be assessed by the goodness of the power-law fit, and how close it is to the critical point by the value of the power-law exponent. Power-law fits to trough cascade size distributions displayed significantly smaller residuals than for peak-cascades at low frequencies between 6 and 34Hz, at 66Hz, and between 104 and 206Hz with an average p-value of .013. This result indicates that trough cascades yielded a better power-law fit than the peak cascades in a low (<35 Hz) and a high frequency band (> 100Hz). As we have argued in the introduction, including both, peaks and troughs as events into the analysis, might bias cascade size and duration distributions. We therefore continued our analysis only with the better fitting trough-cascades.

For the trough cascade size distributions (**Fig 2B**, *upper panel*), we found low residuals (**Fig 2C**, *upper panel*) and near-critical exponents (**Fig 2C**, *lower panel*) that were not statistically different (*p*>.05) from a critical value of α = -1.5 at low (9–37 Hz) and high frequencies (170–275 Hz), as determined by a permutation test. In all other frequency bands α differed significantly from the theoretically assumed critical exponent value of -1.5 demonstrating that networks in these frequency bands did not display near-critical dynamics. We also systematically varied the bin duration used for cascade reconstruction (**Fig 2B**, *lower panel*). Near-critical exponents were only found for longer bin durations (**Fig 2C**, *lower panel*). Shorter bin durations only yielded exponents significantly more negative (supercritical) than the critical value of α = -1.5 across all frequencies.

**Fig 3:**
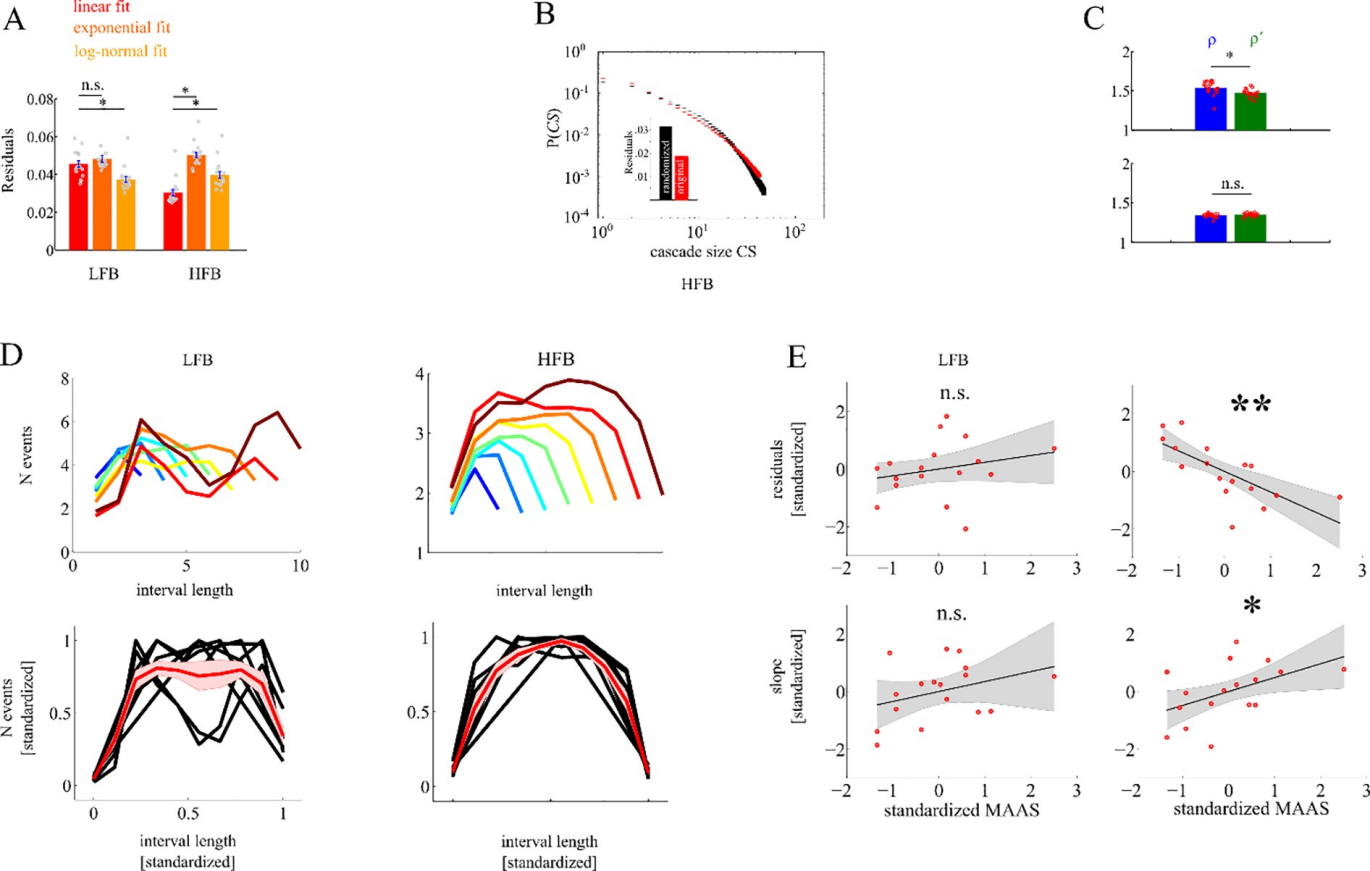
Depiction of cascade size distribution across frequencies. ***A*** We found better linear fits in the HFB compared to exponential and log-normal fits but no such pattern in the LFB. ***B*** We found high residuals of the linear fit to the CS taken from randomized data (black) as compared to empirical data (red). ***C*** ratio of CS and CD slopes were not different from correlation slopes between CS and CD indicating criticality in HFB but not in the LFB. ***D*** shows cascade evolution (shape) as a function of cascade length for both LFB and HFB. Note that only HFB shows comparable cascade shapes for different cascade sizes. ***E*** in the HFB higher MAAS score predicted a better linear fit as indicated by smaller residuals and were also correlated with the slope of the linear regression.

A more detailed analysis of criticality was then carried out in two candidate frequency bands determined from the frequency-specific analysis above, i.e. for a low frequency band (LFB: 9–37 Hz) and a high frequency band (HFB: 170–275 Hz). We chose a bin duration of 10 ms since this is the mean bin duration in previous studies (see for example [9]), and, depending on frequency, shows low residuals of power-law fitting and near-critical exponents in our data. First, we fitted truncated power-laws to the cascade size distributions derived from these two bands. In the HFB, the power-law yielded significantly better goodness of fit than an exponential (**Fig 3A**, res_exp_ = .051, res_lin_ = .031; t_16_ = 16; p < .0001), and a log-normal function (res_lognorm_ = .04; t_16_ = 6; p < .0001), but not in the LFB (res_exp_ = .046; res_lin_ = .049; t_16_ = 1.05; p>.05) where the log-normal fit was better than the linear fit (res_lognorm_ = .038; t_16_ = 3.5; p = .003). Thus, only in the HFB cascade size distributions were better explained by a truly scale-free distribution than by other heavy-tailed distributions. Next, we compared in the HFB the goodness of fit and the estimated exponents with those obtained from surrogate MEG-signals time-shifted across magnetometers, in which the temporal relation across magnetometers was perturbed while the temporal succession of events within each MEG magnetometer was maintained. Residuals of the power-law fits were significantly larger in the HFB surrogate than in the original data (t_16_ = 12.8; *p* < .0001; see **Fig 3B**). This shows that in the HFB cascade sizes better fit to a scale-free distribution than the randomly permuted surrogate data. Residuals in the LFB were generally larger than in the HFB, and did not significantly differ from the residuals of the surrogate data (*t*_16_ = 2.2; *p*>.05). To further evaluate criticality, we tested whether the ratio ρ*´* = $\frac{\alpha -1}{\tau -1}.$ derived from the exponent α of the cascade size distribution (A) and the exponent τ of the cascade duration distribution (B) differed from the exponent ρ of the average size distribution over fixed cascade durations (C) for the LFB and the HFB, respectively. The confidence interval (t_ci_ = ±2.5) was exceeded only in the LFB (t_16_ = 3.8; ρ*´*_LFB_ = 1.53; ρ_LFB_ = 1.47) indicating a significant difference between ρ*´* and ρ for the LFB, but not for the HFB (t_16_ = 2.1; ρ*´*_HFB_ = 1.33; ρ_HFB_ = 1.34; see **Fig 3C**). The observed relation ρ_HFB_*´* = ρ_HFB_ [28] can be regarded as a genuine sign for criticality in the HFB. Criticality for the LFB, instead, could not be verified by this test, as ρ_LFB_*´* ≠ ρ_LFB_.

Scaling of cascades can be directly assessed by analyzing the cascade shapes, i.e. its momentary size as a function of cascade duration (**Fig 3D**). In the HFB all cascades have the shape of a parabola. With different cascade sizes this shape is scaled by a power-law reflecting the underlying scale-free avalanche dynamics. Indeed, in the HFB, all shapes collapse when scaled individually to unit length. This is again not the case for the LFB. Here, cascade shapes are much more variable, and are not simply scaled versions of each other.

To assess the behavioral relevance of our findings, we correlated both the residuals of individual power-law fits as indicators of scale-freeness, and the corresponding exponents as an indicator of criticality with individual MAAS scores of mindfulness across subjects (**Fig 3E**). In the HFB a significant negative correlation was found for the residuals (r = -.71; p = .0015) demonstrating a better goodness of scale-free power-law fits with increasing MAAS score. In parallel, a significant positive correlation was found between individual linear exponents and the MAAS score (r = .48; p = .049). No significant correlations were found for the LFB, neither for the residuals (r = .23; p = .36), nor the exponents (r = .34; p = .17). Altogether, a dynamics showing scale-free, near critical dynamics could be only verified for the HFB.

### Differences between mindful focused attention and rest

**Fig 4:**
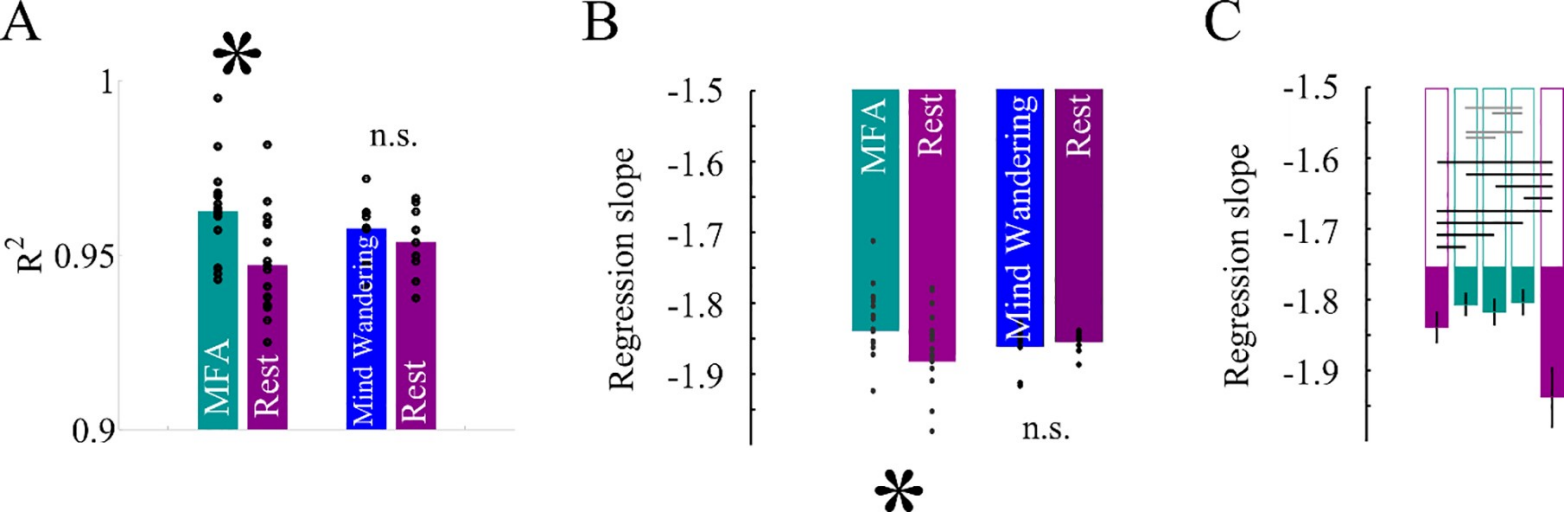
***A*** shows that the linear fit explained almost perfectly variance in the cascade size distributions of both conditions in both groups. ***B*** shows that only the HFB showed a significant difference between blocks in group 1 with CS more closely to α = -1.5 during MFA but not for the control group. ***C*** shows regression slope α for each of the 5 blocks. All black lines indicate statistically significant pairwise differences. The gray lines indicate pairwise comparisons which did not show significant differences.

The previous analysis of criticality was carried out on background MEG pooled across all conditions. We then analyzed the dependence of criticality on our experimental conditions by reconstructing cascade size distributions separately for groups and block conditions in the HFB. **Fig 4A** shows the goodness of power-law quantified by the coefficient of determination for the resting blocks and the MFA blocks in group 1, and for the resting blocks and the MW blocks in group 2. In all conditions coefficients of determination were high (R^2^ > 0.93). Between MFA and rest R^2^ showed a significant difference (t_16_ = 6.6, p < .0001) while there was no such difference between MW and rest (t_9_ = 1.2, p = .25). Nevertheless, our results show that cascade size distributions were scale-free in all conditions. However, as can be seen in **Fig 4B**, the exponent of the power-law was significantly more negative during MW and rest than during MFA (t_16_ = 3.7; p = .002). No difference was found between exponents of the rest and MW condition (t = .65; p = .53). Therefore, with an equally high goodness of power-law fit, power-law exponents were closer to the critical value of α = -1.5 during MFA. Apparently, MFA shifted the dynamics from a supercritical closer to a critical state. Notably, even for the individual experimental blocks in group 1 (**Fig 4C**) we found significantly more negative exponents below -1.5 in the HFB of the resting blocks compared to the 2^nd^ (p = .005), 3^rd^ (p = .02) and 4^th^ MFA block (p = .02), while there was no difference in the exponent across the MFA blocks (all *p* >.13). In contrast, the exponent in the last (5^th^) resting block of group 1 was much more negative than in any other block, including the first resting block (*p*s < .001), and the 2^nd^ (p = .0003), 3^rd^ (p = .0003) and 4^th^ (p = .0008) MFA blocks. No changes were observed across blocks of MFA that could be attributed simply to the passing of time. In contrast, the exponent in the HFB during the last resting block was more negative consistent with a more supercritical regime than in all other blocks, which might indicate fatigue in the course of experiment. This might also explain the slightly reduced goodness of fit in the resting condition particularly of group 1. All this confirms that meditation shifted the scale-free brain dynamics closer to the critical point.

### Topographic distribution of avalanches

**Fig 5:**
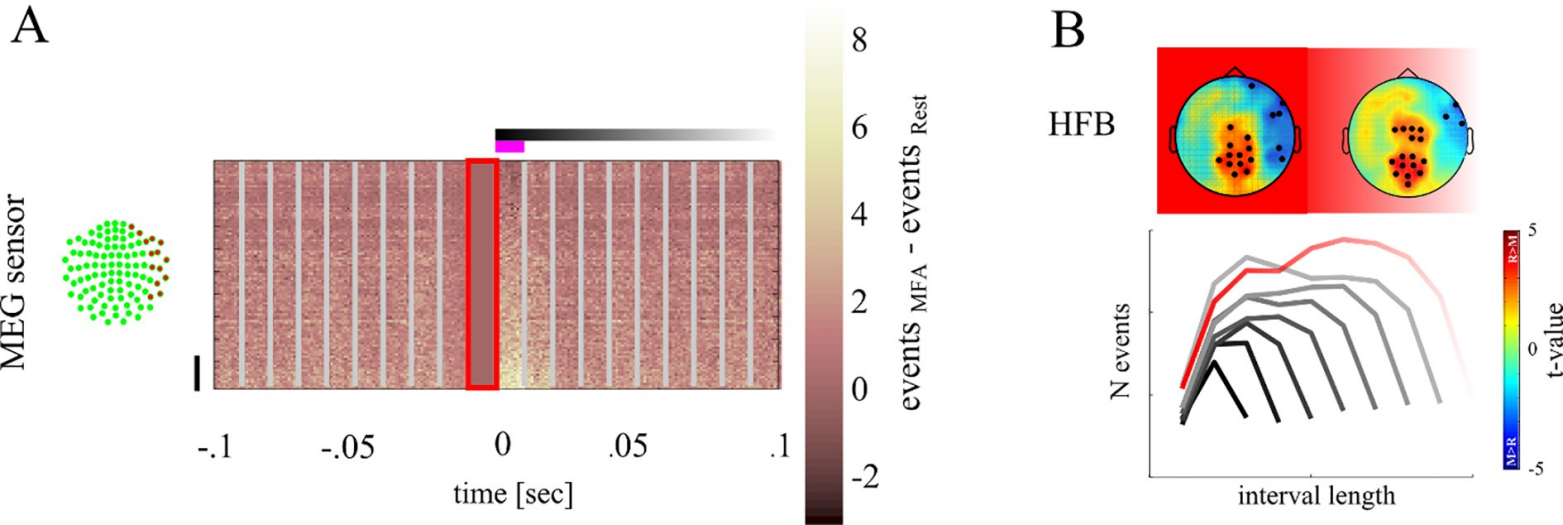
Depiction of topographical distribution differences between conditions. ***A*** shows events centred on start of the cascade as marked by 0 for each magnetometer (y-axis). The red framed area denotes the time bin in which no event was found. In each MEG magnetometer we summed all events found at each sample point both for rest and MFA. Here we depict the difference in the number of events. Light areas indicate that more events were found in the MFA condition while darker areas indicate that more events were found in the Rest condition. The upper panel shows the events found in the HFB. Vertical lines show the temporal bins of 10 msec. MEG magnetometers were sorted according to the number of events found in the first time bin. MEG magnetometers marked by the black vertical line are those showing the strongest difference between rest and MFA and are located over the right hemisphere. This difference is stronger in the HFB than in the LFB. ***B*** shows the topographical distribution of the likelihood of MEG magnetometers to be involved in HFB cascades (left in the first time bin and right across all time bins). Blue areas show regions of MEG magnetometers in which the likelihood is higher during MFA than during rest. Red areas show regions of MEG magnetometers in which the likelihood is higher during rest than meditation. MEG magnetometers at which we observed a significant difference are marked with a black dot.

**Fig 5A** shows the distribution of cascade events as function of time and magnetometer channels relative to avalanche onset. From these spatiotemporal distributions we assessed whether cascades started and/or spread across the same cortical regions during the different experimental conditions. For each magnetometer we determined the likelihood of being member of a cascade either at its start, or during the entire time of its spread. We then compared the regions from which cascades were triggered, or that were traversed by cascades more often in the MFA vs rest condition, respectively. The rationale is that when MEG magnetometer are more often involved at the start or during a cascades at rest compared to MFA, then cascades branch across different regions under these conditions.

**Fig 5B** shows topographical distributions of t-values of the likelihood of magnetometers contributing to rest vs. MFA at the start *(left)* or throughout the entire cascade *(right)* for the HFB, i.e. whether cascades more often started at or spread over a magnetometer during rest (red areas, positive t-values) compared to MFA (blue areas, negative t-values), respectively. Magnetometers with statistically significant t-values compared against the surrogate distribution (t_crit_ = 2.5) are marked by black circles.

MEG magnetometers covering the right fronto-temporal-parietal region triggered HFB cascades more often during MFA (*t*_16_ = -2.8; *p* = .003). A similar pattern was found in the HFB across the entire cascade duration. MEG magnetometers covering the mid-central regions were more often involved in cascades during rest (*t*_16_ = 2.9; *p* = .003), whereas right fronto-temporal-parietal regions were traversed by HFB cascades more often during MFA than during rest (*t*_16_ = -2.6; *p* = .006). In summary, we found that cascades spread differently across cortical regions during rest and MFA. Note, that this does not necessarily mean that cascades were confined to these regions.

## Discussion

Previous analyses on criticality have been carried out on broad-band filtered signals. However, filtering the MEG signal in a broad-band does not necessarily imply that criticality itself is a broad-band phenomenon. In a true broad-band phenomenon, all frequency should similarly contribute to power-law scaling. Our study however shows that peaks and troughs in the broad-band MEG commonly used to reconstruct neural avalanches are phase locked to oscillations in the low (<50Hz) and high (>100Hz), but not in the mid-frequency range. Thus, peaks and troughs are part of spatiotemporally extended oscillatory structures in these two bands. In parallel, power-law fitting yielded low residuals and near-critical exponents in a low (LFB: 9–37 Hz) and a high frequency band (HFB: 170–275 Hz). Thereby troughs showed a significantly better fit in a low and a high frequency band overlapping with LFB and HFB, respectively. Clear evidence for criticality, also, was only found for troughs from MEG signals filtered in the HFB: only in the HFB the goodness of fit to power-law scaling was significantly better than for alternative exponential and log-normal distributions, and also better than for randomly permuted surrogate data. Also the exponent relationship of cascade size and duration distribution proposed by [28] as a genuine sign of criticality, only held for the HFB, but not for the LFB. Furthermore, temporal profiles of avalanches of different size were power-law scaled versions of the same parabolic shape, and could be collapsed onto each other after rescaling. This is regarded as an indicator of criticality, which, however, was only found for the HFB, but not for the LFB. Moreover, only in the HFB, the MAAS score of mindfulness was positively correlated both with the goodness of the power-law fit, and with the exponent of the linear fit across subjects. Our study therefore demonstrates, that criticality in the MEG during MFA is associated with brain oscillations in the high frequency (>100 Hz) range of the MEG.

The frequency dependence of criticality, and the significantly better power-law fit of troughs indicates that peaks and troughs might at least stem partially from different phases of an oscillatory generator, or even from different generators contributing differently to the critical dynamics of the system. In any case, including both, peaks and troughs in the analysis can bias the avalanche size distributions towards larger cascade sizes. By counting peaks and troughs, the contribution of a single dipole, or a single oscillatory generator to an avalanche would be counted twice, and in case of different, superimposed oscillatory generators, avalanches including peaks and troughs would represent a stronger mixture of physiologically different events than peaks or troughs alone. In order to reduce this bias, the analysis of critical brain dynamics on a macroscopic level should take into account the polarity and the frequency of the brain signals. Temporal filtering and selection of troughs thereby can separate the contribution of different generators, and improve the specificity of avalanche reconstruction.

As has been shown, high frequency (>80 Hz) activity in field potentials reflects the feedforward propagation of action potentials more directly than other bands [30]. Thus, high frequency activity would be an ideal carrier of neural avalanches that can spread over the brain revealing the dynamic state of the brain underlying the transmission process.

Importantly, during MFA neural avalanche dynamics reconstructed from the HFB was closer to the critical point compared to MW and rest. While all conditions yielded a similar goodness of fit to a scale free power-law, exponents were closer to the critical value of -1.5 during MFA, and more negative during MW and rest, indicating a shift towards a supercritical dynamics. The largest negative deviation from the critical exponent -1.5 was found in the final resting block most likely due to fatigue.

In our experiment we instructed subjects to breathe normally. However, drawing attention to the breathing cycle as during MFA, breathing might have been altered which could in turn lead to spectral changes. In a recent study, evidence has been found that the breathing rate correlates with slow cortical potentials characterized by oscillations below 1 Hz [31], which is however outside the MEG-bandwidth we investigated and evidence for breathing to alter criticality is lacking. Also, there were no other low frequency changes in the MEG-spectrum of our data that would point towards an altered breathing pattern. The main spectral power effect we found was in a high frequency band (>40 Hz), which could in principle be due to muscle artifacts. As reported in a review by [32], muscle artifacts in the MEG are characterized by broadband high-frequency activity in the range of 20–300 Hz but with the largest power at the lower end of this range. Therefore, changes in muscle activity should have altered lower beta/gamma-power, as well, which was not the case in our data. Furthermore, a significant correlation between high-frequency power reduction and MAAS score was found only unilateral at a right fronto-temporal site. With respect to the initiation and overall contribution to cascades, largest differences between conditions were found at mid-occipital and again at right fronto-temporal sensors. Such topographic distributions would be hard to explain by MFA related changes in muscle activation. Given the additional fact that we rejected muscle-contaminated MEG signals without noting differences between experimental conditions, a mere muscle effect on criticality is unlikely. Notably, the critical dynamics we observed especially in the high frequency band corresponds to a lower signal power in this band. Hence, we would conclude that at least critical dynamics was not driven by muscle artifacts. If any, reconstruction of avalanche dynamics could have been affected by muscle artifacts during resting state conditions. In this case, MFA would have facilitated the detection of critical brain dynamics by reduction of muscle activity.

However, given the fact that both the goodness of fit of the power-law and the power-law exponent were correlated with the MAAS score rather suggests that the shift of the exponent towards criticality during MFA was truly related to a change in brain dynamics. We used the MAAS score as independent measure of the subject's tendency toward mindfulness, a trait of the subject that is not related to the actual performance during the experiment. Notably, subjects did not differ as to their trait level of mindfulness between the MFA and the MW group, which shows that group differences in critical dynamics was related to the instruction, and not to differences in the subject's meditative capabilities. To our knowledge, there are no well-established psychophysiological or behavioral markers that specifically quantify such a state without being confounded by unspecific effects like vigilance. We therefore thoroughly instructed our subjects to perform meditation without changing breathing, and explicitly used naïve subjects to show the differences between rest and mediation. To control for vigilance effects, our experimental design used blocks of rest in the beginning and at the end. Under the assumption of drowsiness we would have expected that brain dynamics alters progressively across the entire experiment. Indeed, a vigilance effect was found in the meditation group. Between the first and the second resting state at the end of the experiment, the cascade size exponent became more negative than -1.5 indicating a shift towards supercritical behavior. This might be an effect of drowsiness. But this was only found in the resting state blocks, whereas no significant change occurred across meditation blocks. Also, we did not find differences in alpha band power indicating a vigilance effect, and our experiment was relatively short (~30 min in total) reducing the influence of vigilance decrements.

A few other studies have also analyzed the relationship between meditation and criticality. Irrmischer et al. [16] showed a reduction of long range temporal correlations (LRTCs) during MFA. They hypothesized that attention is balanced at a critical point of instability between order and disorder allowing for both, transient focus and swift change of attentional resources. They argue that the focus of attention reduces information propagation by shifting the system towards a subcritical regime, which seems to be in contrast to our findings. However, consistent with our study, the authors did not find effects of MFA on critical brain dynamics in novices at frequencies < 45 Hz. Also, in our study, the power-law exponent changed from values more negative than -1.5 to values close to -1.5, i.e. in a similar direction from a supercritical to a critical dynamics. Furthermore, differences between our study and Irrmischer et al. [16] might arise from difference in measurement (MEG vs. EEG) and analysis (various avalanche based indices of criticality vs. detrended fluctuation analysis, DFA). Thereby, it is not straight forward to relate DFA to the power-law distributions and criticality indices used in our analysis. Most importantly, their study did not include higher frequencies from 170 to 270 Hz as our study and their effects were only found in trained subjects. Fagerholm et al. [10] found that in the resting state broad-band cascades were associated with an approximate critical power-law form, while the focused task state was associated with a subcritical dynamics. This parallels our study insofar as critical dynamics were found when subjects are instructed to focus on their internal milieu and refrain from the external world. Our study adds an important point to the knowledge of criticality since we showed that critical dynamics are driven by high frequency activity which was not assessed in previous EEG studies. Notably, Fagerholm et al. [10] focused on stimulus-driven attention rather than on self-regulated modulation of top-down attention as in MFA. Also, subjects had to respond as fast and accurately as possible, which necessarily confounds brain dynamics underlying attention with stimulus-evoked responses, visual information processing, decision processes, and movement-related activation. Hence, subcritical dynamics could not be ascribed exclusively to attentional modulation. In general, motor tasks that require decisions under focused attention are not the best candidates to contrast with rest, especially since fluctuation between stability and instability as the identifying feature of criticality is suspended during motor tasks, leading to increased stability [33]. Hence, studies on deviation from or convergence to criticality during attention must control for motor activity, visual input and decision processes. Finally, while Fagerholm et al. [10] proposed that the distribution of cascades changes with different cognitive states rather than where their origin, we found that cascades during MFA were triggered more often in the right hemisphere. Our finding thereby is consistent with several studies showing that during top-down attention right-hemispheric regions play a prominent role [34,35]. Unlike in our study, Lutz et al., while recording EEGs in long-term Buddhist meditation practitioners, observed in the gamma-band high amplitude oscillations and phase synchrony between fronto-parietal electrodes at frequencies from 25 Hz to 42 Hz, i.e. at lower frequencies than the high-frequency effects in our study [36]. The differences between Lutz et al. and our findings may be due to different styles of meditation, since Lutz et al. aimed at a state of non-referential compassion meditation, for which a focused attention on a particular object, was not required.

In our study, we asked the subjects to rest in the first and last block and to perform MFA in the intermittent blocks. The instruction to rest allows the mind to wander [37]. Mind wandering is characterized by a lower level of alertness [38], and reduced external information processing, which could also explain the larger distance from a critical state, as is the case under rest, particularly if the subjects get tired. Moreover, during mind wandering attention is assumed to be decoupled from the environment [38]. By contrast, MFA requires the detection and inhibition of mind-wandering, and the refocusing of breath, which involves constant monitoring and executive control, particularly in novices. Clearly, self-regulated top-down modulation of attention as required during MFA is not well described by a fixed filter with a narrow aperture. This process is better characterized by a balance of noise and stability, of integration and segregation, of excitation and inhibitions, as might be implemented by brain states close to an instable critical point at the border between order and disorder. The observed decrease in power of the high-frequency activity during MFA in our study might thereby reflect a lower rate of neuronal spiking in right fronto-parietal regions during MFA, and thus a reduced destabilizing drive, "distracting" the brain from its balanced critical state during mind-wandering.

It is usually assumed that during rest the Default Mode Network (DMN) is activated, whereas studies of focused concentrative mediation have reported fronto-parietal executive network activity during meditation [39]. Particularly, as shown by functional magnetic resonance imaging (fMRI), MFA is correlated with activation of prefrontal cortex, premotor cortex, and dorsal anterior cingulate cortex that have been also shown to be involved in self-regulation of attention [40], as well as a reduced activation of posterior cingulate cortex, and the posterior parietal lobule of the DMN that have been related to mind-wandering, before [39,41].

In conclusion, we have shown that criticality as obtained from avalanches in MEG recordings was only observed at high frequencies > 100 Hz, and that during mindful focused attention avalanche dynamics was closer to a critical point than during states of rest. Together with the finding that mindfulness meditation leads to topographic changes in the avalanches relative to rest, our results show that self-regulated attention as required during meditation tunes brain dynamics to criticality providing a functional link between criticality and cognition.

## Supporting information

S1 Data Anleitung meditation. (DOCX)
