# Supplementary material for: Self-regulated critical brain dynamics originate from high frequency-band activity in the MEG
Source: PLoS One. 2020 Jun 11;15(6):e0233589. doi: 10.1371/journal.pone.0233589 (PMC7289413; doi:10.1371/journal.pone.0233589)
Supplement: S1 Data — (DOCX) [file pone.0233589.s001.docx]

Anleitung Meditation

Part 1:

Ich werde jetzt eine kurze Meditation anleiten mit dem Atem als primäres Objekt der Wahrnehmung.

Setz Dich dafür einfach in eine Position, die möglichst gemütlich für Dich ist. Eine, in der der Kopf ausbalanciert auf den Schultern sitzt und Hände und Arme in einer entspannten Lage ruhen. Dies ist nun eine kurze Zeit, in der Du Dir erlauben kannst von Deinem normalen Modus des Tuns, der Bewegung und des Reagierens in einen Modus des einfachen Seins zu wechseln. Einen Modus, in dem man einfach aufmerksam ist, was im Inneren in einem Selbst passiert. Genau hier und genau jetzt.

Und nun während Du sitzt, beachte einfach das Gefühl des Atems, fühle wie sich Dein Bauch bewegt mit jedem Ein- und jedem Ausatmen. Die Bewegung der Luft durch Deine Nasenlöcher, die schwache Bewegung Deiner Brust und Schultern. Bringe Deine Aufmerksamkeit zu dem Part des Atemzyklus, der sich in Deinem Körper am intensivsten anfühlt. Und bemerke den gesamten Kreislauf des Atems. Angefangen mit dem Gefühl der Luft, die in Deinen Körper einströmt, Deine Lungen füllt und leicht Deinen Bauch hebt. Die Bewegung der Luft, die hinausströmt. Und mach Dir die Pause dazwischen bewusst, der Moment der Pause zwischen Ein- und Ausatmen. Zwischen Ausatmen und dem nächsten Einatmen. Das alles ist eine Bewegung, ein Kreislauf.

Part 2:

Und Du wirst bemerken, dass sich Deine Aufmerksamkeit von Zeit zu Zeit verlagert. Weg von Deinem Atem. Deine Gedanken wandern vielleicht zu Fantasien oder Erinnerungen, Gedanken über den Tag, Sorgen, die Du möglicherweise hast, Dinge, die Du erledigen musst. und ohne es Dir selbst schwer zu machen, wenn Du bemerkst, dass das passiert, bringe einfach sanft aber bestimmt Deine Wahrnehmung zurück zu dem Gefühl des Atmens, der sich durch Deinen Körper bewegt.

Part 3

Und es ist möglich, dass wenn Dein Bewusstsein gerade woanders ist, das Flackern der Aufmerksamkeit zu greifen, der Moment, in dem Du realisierst, dass Dein Bewusstsein gerade woanders ist als beim Atem, ist ein Moment der Erkenntnis. Und wenn Du Dir bewusst bist, dass Deine Gedanken gerade wandern, bring ganz einfach Deine Aufmerksamkeit zurück zu Deinem Atem.

Part 4

Und nun, da die Meditation zu einem Ende kommt, vergenwärtige Dir, dass Du eine Zeit verbracht hast, in der Du Dir Deiner Moment-zu-Moment Erfahrung bewusst warst. Dass Du Deine Kapazität erweitert hast, Deine Sinne zu erleben, in dem präsenten Moment. Und wenn Du bereit bist, dann beende diese Meditation, öffne Deine Augen und bewege Deine Gliedmaßen, wenn Du magst. Schließe danach Deine Augen wieder und verbleibe noch zehn Minuten in einem entspannten Zustand.

Part 1:

I will now initiate a short meditation with the breath as the primary object of perception.

Just sit in a position that is as comfortable as possible for you. One in which the head is balanced on the shoulders and hands and arms rest in a relaxed position. This is now a short time in which you can allow yourself to switch from your normal mode of doing, moving and reacting to a mode of simple being. A mode in which you are simply attentive to what is happening inside of you. Right here and right now.

And now as you sit, just pay attention to the feeling of the breath, feel your abdomen move with each inhalation and each exhalation. The movement of the air through your nostrils, the gentle movement of your chest and shoulders. Bring your attention to the part of the breathing cycle that feels most intense in your body. And notice the entire cycle of breathing. Beginning with the feeling of air flowing into your body, filling your lungs and gently lifting your abdomen. The movement of the air that flows out. And notice the pause in between, the moment of pause between inhaling and exhaling. Between exhalation and the next inhalation. All this is a movement, a cycle.

Part 2:

And you may notice that your attention shifts from time to time. Away from your breath. Your thoughts may wander to fantasies or memories, thoughts about the day, worries you may have, things you have to do. And without making it hard for yourself, when you notice that this happens, just gently but firmly bring your perception back to the feeling of breathing moving through your body.

Part 3

And it is possible that when your consciousness is somewhere else, to grasp the flickering of attention, the moment you realize that your consciousness is somewhere else focused on the breath, is a moment of realization. And when you are aware that your thoughts are wandering, just bring your attention back to your breath.

Part 4

And now that the meditation is coming to an end, realize that you have spent a time being aware of your moment-to-moment experience. That you have expanded your capacity to experience your senses in the present moment. And when you are ready, end this meditation, open your eyes and move your extremities if you wish. Then close your eyes again and stay in a relaxed state for another ten minutes.
